# Cardiomyopathy: A New Perspective from Diagnostic Strategy

**DOI:** 10.3390/jcm12062360

**Published:** 2023-03-18

**Authors:** Keiichi Hirono

**Affiliations:** Department of Pediatrics, University of Toyama, Toyama 930-0194, Japan; khirono@med.u-toyama.ac.jp; Tel.: +81-76-434-7313

Cardiomyopathy entails a broad group of diseases, acquired or genetic, which result in a similar phenotype. Furthermore, cardiomyopathy is a clinically heterogeneous disease with large differences in gender, age of onset, and rate of progression, which are thought to be explained by a complex interplay of genetic susceptibility and environmental factors [1,2]. The causes of cardiomyopathy can be categorized as genetic or acquired, but the two are not mutually exclusive. The influence of environmental factors may be added to a genetic predisposition, and a disease phenotype may emerge. Interestingly, the pathogenesis of Takotsubo cardiomyopathy is that the autonomic sympathetic nervous system is excited, resulting in the strong stimulation of cardiac myocytes via norepinephrine and catecholamines in the blood secreted by the adrenal glands, which damage the myocardium [3]. Phenotypes often differ because of various environmental factors superimposed onto a genetic predisposition. Under these circumstances, the MOGE (S) classification was proposed as a method of expressing morphofunctional phenotype (M), organ and systemic involvement (O), genetic pattern (G), etiology (E), and stage and severity (S) in an integrated manner. This classification has attracted attention as a new diagnostic method that can identify disease risk and severity over time and select a more appropriate treatment. This classification is attracting attention as a new diagnostic method that allows for the risk and severity of disease to be determined over time and more appropriate treatment to be selected. For example, we showed that left ventricular noncompaction (LVNC) is associated with congenital heart disease, ranging from patent ductus arteriosus or atrial septal defects/ventricular septal defects to more severe diseases such as Ebstein’s disease [4]. We also reported a pediatric case of LVNC, atrial septal defect, atrioventricular conduction disorders, and syncope caused by a novel variant in the *NKX2-5* gene [5]. Thus, the MOGE classification provides a clearer understanding of this phenotype.

Because of the wide variety of conditions that can lead to cardiomyopathy, a systematic approach is needed to facilitate the identification and management of specific cardiomyopathies. The diagnosis, management, and follow-up of patients with cardiomyopathy is a multifactorial process [6]. Although LVNC has received increasing attention over the past two decades, LVNC is still rarely considered in routine clinical practice. There are no generally accepted diagnostic criteria at present, and some groups do not recognize it as a distinct cardiomyopathy and even grade it as a variant of dilated cardiomyopathy or a morphological feature of a different condition. Arrhythmogenic cardiomyopathy (ACM) is a new disease that has been underestimated in the past. Clinical, electrocardiographic, and arrhythmogenic features should raise diagnostic suspicion and prompt a more detailed examination, even when echocardiography is normal [7]. The clinical features of patients with arrhythmogenic left ventricular cardiomyopathy include frequent myocarditis-like episodes, significant electrical instability, and the need for frequent implantable cardioverter defibrillator (ICD) implantations [8].

Multimodality imaging is an important screening tool for the identification of various cardiomyopathies and is often the first cause of clinical suspicion of a specific etiology, especially when the history and familiarity of the disease is vague [9]. Multimodality imaging with different imaging techniques, such as echocardiography, cardiac magnetic resonance, cardiac-computed tomography, and nuclear cardiac imaging, provides essential information for diagnosis, the stratification of sudden cardiac death, and management. Echocardiography is noninvasive and reproducible, and can detect various cardiomyopathy phenotypes, suggesting the cause of disease, cardiac morphology and hemodynamics, and disease severity. It plays a fundamental role in all the necessary steps for management and treatment strategies in clinical practice. Speckle Tracking Echocardiography (STE) can detect subclinical ventricular contractile function in early heart disease with normal left ventricular ejection fraction. The left ventricular Global Longitudinal Strain obtained by STE is the most- used parameter in clinical practice. Myocardial workload corrects STE-derived parameters for afterload using systolic blood pressure. Cardiac magnetic resonance is the second most valuable imaging modality for the differential diagnosis of physiology and pathology. It is highly accurate and reproducible, providing information on ventricular morphology, function, and magnetic properties beyond the presence and localization of fibrosis. The evaluation of these parameters is useful in a variety of settings, including diagnosis, risk stratification, and differential diagnosis. Cardiac computed tomography (CCT) shows high accuracy in the assessment of the origin, course, and termination of coronary arteries. Therefore, the main reason for performing coronary CT angiography is to exclude coronary artery abnormalities and atherosclerotic coronary artery disease.

In recent decades, our understanding of the genetic alterations that give rise to the different phenotypes of cardiomyopathy has advanced dramatically. More than 1000 mutations have been identified in various genes, and different genetic alterations or combinations of genetic alterations have been shown to cause either HCM, DCM, LVNC, or ACM. Recently, mutations in the same gene have been found in several cardiomyopathies with different and possibly overlapping phenotypes of cardiomyopathy [10]. The results of recent studies indicate that common genetic variation plays an important role in cardiomyopathies’ development and progression.

A wide range of imaging modalities, combined with other clinical information and genetics, is useful in the evaluation of cardiomyopathy. Further evidence is needed to improve the diagnostic capabilities of multimodality and make it part of the standard diagnostic workup.

## Data Availability

The author confirms that the data supporting the findings of this study are available within the article.
